# Successful Treatment with Corticosteroids in an 11-Year-Old Patient with Crohn’s Disease and Myopericarditis—Case Report

**DOI:** 10.3390/pediatric14010005

**Published:** 2022-01-11

**Authors:** Joanna Ryzko, Katarzyna Zdanowicz, Dariusz Marek Lebensztejn, Urszula Daniluk

**Affiliations:** 1 Department of Pediatrics, Gastroenterology, Hepatology, Nutrition and Allergology, Medical University of Bialystok, Waszyngtona St. 17, 15-274 Białystok, Poland; joannaryzko@gmail.com (J.R.); lebensztejn@hoga.pl (D.M.L.); urszula.daniluk@umb.edu.pl (U.D.); 2 Department of Gastroenterology, Hepatology, Feeding Disorders and Pediatrics, The Children’s Memorial Health Institute, 04-730 Warsaw, Poland

**Keywords:** Crohn’s disease, extraintestinal manifestations, myocarditis, children

## Abstract

Extraintestinal manifestations (EIMs) are observed in 15–20% of patients with inflammatory bowel disease (IBD). One of the rare EIMs is myocarditis, the incidence of which is estimated at around 1%. The main cause of myocarditis is a viral infection. Other causes include autoimmune diseases and drug complications (sulfasalazine, mesalazine). We present the case of an 11-year-old girl with Crohn’s disease (CD) with EIMs, manifested as hip joint inflammation and erythema nodosum. At the same time, the symptoms of myopericarditis appeared with changes in electrocardiogram (ECG), echocardiography and high troponin I concentration. Therapy with corticosteroids resulted in the resolution of skin lesions and cardiological symptoms. Systemic connective tissue diseases, viral and bacterial infections were excluded in the differential diagnosis. The suspicion of mesalazine-induced EIMs was also ruled out as the symptoms resolved despite continued therapy with mesalazine. No further recurrences of myopericarditis were observed.

## 1. Introduction

Crohn’s disease (CD) is a chronic inflammatory bowel disease (IBD) of unknown etiology, characterized by periods of remission and exacerbation. The pathogenesis of the disease has not been fully understood, and so far genetic, environmental and immunological factors have been mentioned as causes of inflammation.

Myocarditis is an inflammatory process in which myocyte necrosis can occur. The most common causes of myocarditis in children are viral infections (mainly enteroviruses), followed by bacterial infections, drugs, and autoimmune disease. The prevalence of myocarditis concerns about 1% of hospitalized children and about 8–21% of sudden cardiac deaths in children and young adults [1,2].

Myocarditis and myopericarditis may also be a rare extraintestinal manifestation (EIM) of IBD. In most cases it is a side effect of IBD treatment, mainly with sulfasalazine or mesalazine [3,4,5,6,7,8,9,10]. It is estimated that less than 1% of children and adults with IBD present cardiovascular symptoms.

## 2. Case Report

An 11-year-old girl was admitted to the hospital due to pain in the area of the left buttock and hip joint accompanied by a fever lasting several days (up to 39 °C). In addition, the girl reported the occurrence of loose stools for 3–4 weeks and weight loss (about 3 kg). Family history revealed asthma and celiac disease in the patient’s sister. Laboratory tests showed an increase in the concentration of the C-reactive protein (CRP)—49.46 mg/L, low levels of iron (18 μg/dL) and vitamin D3 (12 ng/mL), high fecal calprotectin (FC) (1100 μg/g) and a positive fecal occult blood test. Imaging examination (X-ray, ultrasound) and orthopedic consultation allowed to exclude hip arthritis. An antibiotic (amoxicillin with clavulanic acid), anti-inflammatory drugs and vitamin D supplementation were recommended.

After next 2 weeks, the girl was admitted to our department because of bloody stools and persistent pain in the left hip. Physical examination revealed a lean body posture (BMI 3–10 percentiles), palpable abdominal pain in the lower abdomen, and persistent pain in left hip. Laboratory tests showed increased inflammatory markers such as CRP (60.07 mg/L), white blood count (WBC, 11.90 × 10^3^/μL), erythrocyte sedimentation rate (ESR, 64 mm/h) and a high FC value (2482.7 μg/g). Ultrasound examination of the abdominal cavity revealed a slightly thickened cecum wall (3.7 mm). During hospitalization, bacterial (including tuberculosis), viral (cytomegalovirus, rotavirus, adenonovirus) infections, parasitic infestation and celiac disease were excluded. Gastroduodenoscopic examination revealed scattered aphtous erosions in the antrum of the stomach and erosions covered with fibrin on the anterior and posterior wall of the duodenal bulb. Ileocolonoscopy revealed numerous aphthous erosions in the large intestine and single ones in the tip of the ileum. MR enteroclysis scans showed a thickened wall of the distal ileum. Histopathological examination of the ileum and colon specimens showed the inflammatory lesions suggesting CD. Based on clinical, endoscopic and imaging examinations, CD was diagnosed (Paris classification: A1c, L2, B1 G1). Disease activity based on the PCDAI (Pediatric Crohn’s Disease Activity Index) was rated at 45 points (moderate activity). Exclusive enteral nutrition was started as the first-line therapy for CD. After disease remission was induced, an immunosuppressant drug (azathioprine) and an anti-inflammatory drug (mesalamine) were used to maintain remission.

**Figure 1:**
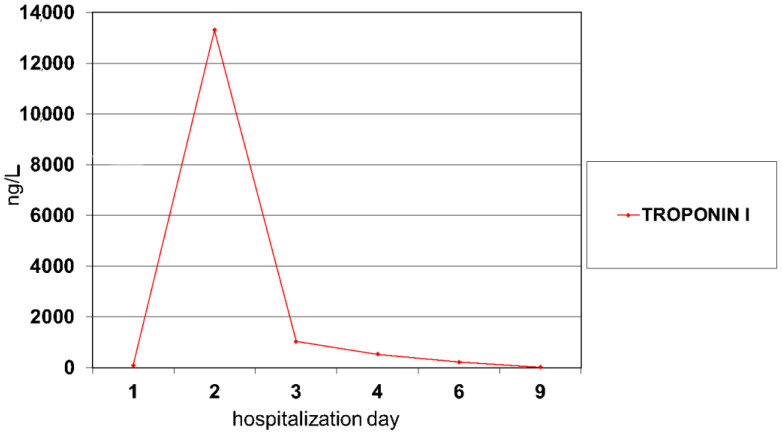
Troponin I values during myocarditis treatment.

Two weeks after the discharge from the hospital, the patient was re-admitted due to deterioration of her general condition with fever, chest pain and erythema nodosum. Using physical examination, tachycardia (HR 140/min), nodular lesions on the extensor of the lower straight parts and underweight (BMI < 3) were detected. Laboratory tests showed a significant increase in inflammatory markers (CRP 234.84 mg/L, WBC 17.1 × 10^3^/µL, ESR 88 mm/hr), a low iron level (8 μg/dL) and high FC (2497.7 μg/g). Due to the reported chest pain, the concentration of troponin was assessed, showing its elevation (92 ng/L [normal value: 0–19 ng/L], Figure 1).

**Figure 2:**
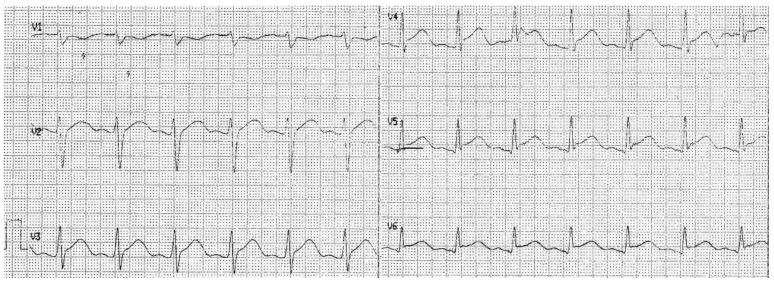
ECG recording on the 3rd day of hospitalization.

The electrocardiogram (ECG) recording was normal. Broad-spectrum antibiotic therapy (amikacin, cephtazidim) and antifungal drugs were used in the treatment. Control tests performed on the second day of hospitalization revealed a significant increase in troponin I (13,312 ng/L, Figure 2), a slight increase in CRP compared to the previous day, hypoalbuminemia (3.17 g/dL) and increased activity of creatine kinase (CK; 297 IU/L) and creatine kinase—MB (CK-MB; 50 IU/L). The ECG record showed an elevated ST segment in V2–V6 leads (Figure 2).

**Figure 3:**
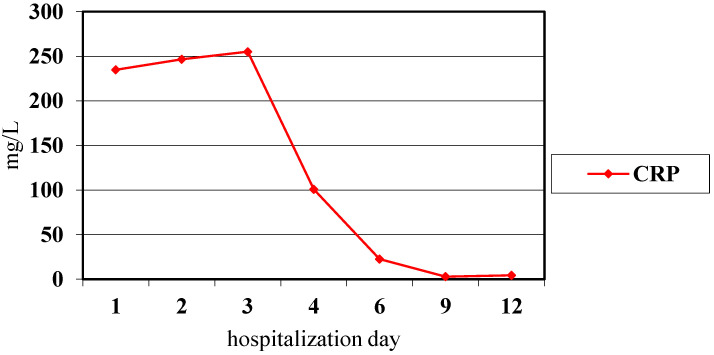
CRP values during observation of myocarditis.

Echocardiography (ECHO) examination did not reveal any significant abnormalities. Vancomycin and systemic steroid therapy (methyloprednisolone) were added to the treatment. On the 3rd day of hospitalization, a gradual decrease of troponin I (Figure 1) and CRP (Figure 3) was noted. ST-segment elevation was still observed in all leads of the ECG record, while ECHO examination revealed the appearance of pericardial effusion with a 5 mm of pericardial separation in diastole. Hyperkinetic circulation was suspected based on increased cardiac output and a high heart rate. Cardiac magnetic resonance imaging (CMR) was not performed because of technical limitations. Due to tachycardia, no signs of heart failure and the need to prolong diastole, a beta-blocker was included in the treatment (metoprolol 23.75 mg/daily). Viral and bacterial infections (Epstein-Barr virus, Cytomegalovirus, Coxsackie, Parvo-virus B19, Influenza A and B, Streptococcus, Mycoplasma, Yersinia) were excluded in the differential diagnosis of myopericarditis. Serological tests for systemic connective tissue diseases were negative. In subsequent control tests, a systematic improvement in the patient’s general condition was observed with a gradual decrease in the level of troponin I and inflammatory markers (Figure 1 and Figure 3). The girl was discharged home with the recommendation to continue steroid therapy with gradual dose reduction. Due to the high resting heart rate (90–110/min), cardiac therapy was extended to 12 months. CD treatment with azathioprine and mesalazine was continued.

After 2 years of combination therapy with azathioprine and mesalazine, another exacerbation of CD with loose stools (up to 4–5 per day), abdominal pain, low-grade fever and pain complaints of elbow and knee joints was observed. Laboratory results showed an increase in inflammatory parameters (ESR 43 mm/h, platelets 498,000/µL) and mild anemia (Hb 11 g/dL). FC increased to 2527.5 μg/g. The endoscopic examination revealed disseminated mucosal aphthous erosions in the descending part of the duodenum and extensive inflammatory changes along the entire length of the colon, as well as single aphthous erosions in sigmoid and rectum (Paris classification—A1b, L2, B1). The PCDAI score was assessed at 55 points, which corresponds to a severe disease. Taking into account the disease progression, biological treatment with adalimumab was started. Azathioprine treatment was continued. During the 3-year monitoring conducted by cardiologist, no recurrences of myopericarditis or complications after myopericarditis were found.

## 3. Discussion

In the presented case, CD was diagnosed based on the manifested symptoms (abdominal pain, weight loss, recurrent diarrhea with negative stool cultures), endoscopic findings (inflammatory lesions in the terminal ileum and caecum and stomach), histopathological examination, and the image of MR enteroclysis (thickened wall of the distal ileum). It is worth to notice the gradual progression of the clinical course from nonspecific abdominal pain and diarrhea at the onset of the disease to extraintestinal symptoms of CD with cardiac involvement [4].

However, cardiac involvement in the course of IBD is very rare. In the literature, which mainly concerns adults, a higher incidence of myocarditis is noted in patients with ulcerative colitis (UC) than CD [11]. The pathomechanism of myocarditis in IBD seems to be more complex and not fully understood [12]. The coexistence of infectious, autoimmune conditions or drug-related complications are considered to be involved in development of miocarditis. In the presented case, the viral background was excluded on the basis of serological tests. There was also no indication for an endomyocardial biopsy according to the guidelines of The American College of Cardiology, the American Heart Association and the European Society of Cardiology [13,14].

Due to rapid improvement after initiation of corticosteroids with concurrent resolution of intestinal and skin lesions and resolution of clinical and biochemical symptoms of myocardial disease, cardiovascular disease should be treated as an EIM of CD, similar to erythema nodosum and hip symptoms in our patient. McGrath-Cadell et al. reported a case of a young women with mobile valvular masses and myocarditis possibly secondary to active CD, in which clinical improvement was not achieved despite treatment with broad-spectrum antibiotics. Only after administration of steroids (prednisolone) and anticoagulants the symptoms were finally relieved [15]. However, in another report, acute myopericarditis occurred 2 weeks after the intensification of therapy (prednisone and mesalazine) due to exacerbation of CD. Clinical improvement was achieved after use of colchicine with an increase in the dose of steroid [16]. Additionally, in adult patients with UC complicated by myocarditis, administration of corticosteroids led to symptoms relief [17,18,19]. The authors of the reported cases proposed the concomitant administration of colchicine to prevent the recurrence of myocarditis [17,18,19].

Some authors have suggested a link between myocarditis and the use of mesalazine [17,20]. Several mechanisms of masalazine toxicity have been proposed, i.e., inhibition of prostaglandin production (inhibition of cyclooxygenase activity), hypersensitivity reactions (cytokines stimulated by eosinophils), cross-reactions of anti-mesalazine antibodies with the cardiac tissue, direct toxic effects of mesalazine on the heart muscle and pericardium, and allergic reactions [18]. In contrast, other authors found no association of mesalazine in the development of myocarditis in IBD patients [12,20,21]. In our case, the relationship between myocarditis and 5-ASA treatment was excluded, because myocarditis did not recur despite continued use of this drug.

In the two-year follow-up of our patient, no myocarditis was observed despite the progression of Crohn’s disease during immunosuppressive treatment requiring the initiation of biological therapy with an anti-TNFα monoclonal antibody. However, the patient requires monitoring of the cardiovascular system, as the literature also includes reports of myocarditis after biological treatment [22,23].

## 4. Conclusions

The reported case concerns the rarely observed coexistence of myocarditis with CD. Systemic steroid therapy in IBD with concomitant myocarditis as an EIM remains the first-line therapy. Due to possible recurrences of myocarditis in the course of IBD, the patient should be under the care of a cardiologist.

## Data Availability

Data available via email.
